# Effects of a Standardised Medical–Dental Collaborative Protocol on Acute Stroke Rehabilitation: A Multicentre Prospective Cohort Study

**DOI:** 10.1111/joor.70143

**Published:** 2026-01-02

**Authors:** Yu Yoshizumi, Yoshiyuki Sasaki, Motoki Inaji, Jun Karakama, Ayako Nakane, Chiaki Matsubara, Junichi Furuya, Shinsuke Irie, Shinichi Wakabayashi, Masateru Katayama, Katsuhiko Sakai, Takanori Hayakawa, Yoshihisa Kawano, Nobuhiro Inokuchi, Manabu Ishihara, Hideyuki Takano, Nobukazu Komoribayashi, Yasushi Tamada, George Umemoto, Kazuyuki Matsunaga, Junichi Yamazoe, Haruka Tohara, Taketoshi Maehara

**Affiliations:** ^1^ Department of Dysphagia Rehabilitation Graduate School of Medical and Dental Sciences, Institute of Science Tokyo Tokyo Japan; ^2^ Oral Surgery, Saitama Red Cross Hospital Japanese Red Cross Society Saitama Japan; ^3^ Clinical Dental Research Promotion Unit, Faculty of Dentistry Institute of Science Tokyo Tokyo Japan; ^4^ Department of Neurosurgery, Graduate School of Medical and Dental Sciences Institute of Science Tokyo Tokyo Japan; ^5^ Department of Neurosurgery Ome Medical Center Tokyo Japan; ^6^ Dentistry and Oral Surgery Japan Community Healthcare Organization Tokyo Shinjuku Medical Center Tokyo Japan; ^7^ Department of Dental Hygiene University of Shizuoka, Junior College Shizuoka Japan; ^8^ Department of Oral Function Management, Graduate School of Dentistry Showa Medical University Tokyo Japan; ^9^ Department of Neurosurgery Kushiro Kojinkai Memorial Hospital Hokkaido Japan; ^10^ Department of Neurosurgery Suiseikai Kajikawa Hospital Hiroshima Japan; ^11^ Department of Neurosurgery, Tokyo Dental College Ichikawa General Hospital Chiba Japan; ^12^ Department of Oral Medicine and Hospital Dentistry Tokyo Dental College Chiba Japan; ^13^ Department of Neurosurgery National Hospital Organization Disaster Medical Center Tokyo Japan; ^14^ Department of Gerodontology and Dysphagia Rehabilitation JA Toride Medical Center Ibaraki Japan; ^15^ Emergency and Critical Care Medicine Tokushima University Hospital Tokushima Japan; ^16^ Department of Oral Medicine, Institute of Health Biosciences The University of Tokushima Graduate Faculty of Dentistry Tokushima Japan; ^17^ Iwate Prefectural Advanced Critical Care and Emergency Center Iwate Medical University Iwate Japan; ^18^ Gerodontology, Department of Oral Health Science, Faculty of Dental Medicine Hokkaido University Hokkaido Japan; ^19^ Swallowing Disorders Center Fukuoka University Hospital Fukuoka Japan; ^20^ Department of Neurology Brain Attack Center Ota Memorial Hospital Hiroshima Japan; ^21^ Section of Geriatric Dentistry and Perioperative Medicine in Dentistry Kyushu University Hospital Fukuoka Japan

**Keywords:** aspiration pneumonia, deglutition, multicentre study, propensity score, prospective studies, stroke

## Abstract

**Background:**

Acute stroke often causes complications such as aspiration pneumonia, which can be prevented through oral hygiene. However, evidence supporting individualised oral care remains limited. We developed a multidisciplinary oral function management protocol and evaluated its benefits in patients with acute stroke, including its impact on aspiration pneumonia.

**Methods:**

In this prospective multicentre cohort study, data from 1616 patients with acute stroke admitted to participating hospitals between 31 July 2017, and 27 January 2021 were analysed. Hospitals implemented our oral function management program (Protocol group) or provided conventional oral care (control group). To minimise confounding by baseline severity, propensity scores were estimated using stroke severity and initial complications; patients were matched 1:1 using nearest‐neighbour matching with a calliper of 0.2, resulting in 313 matched pairs (*n* = 626). Primary outcome was the incidence of aspiration pneumonia; secondary outcomes were hospital stay length and oral function improvement.

**Results:**

No significant differences in aspiration pneumonia onset rates were detected between the protocol and control groups (*p* = 0.7639). Compared to controls, the protocol group showed significant improvements in oral hygiene (*p* < 0.0001), tongue mobility (*p* = 0.0292), and FOIS scores (*p* = 0.0005). Time from admission to discharge evaluation (*p* < 0.0001) and total length of hospital stay (*p* = 0.0018) were significantly reduced. Compared to administration of conventional oral care, implementation of this Protocol facilitated earlier oral management and feeding/swallowing rehabilitation, potentially reducing hospital stays and enhancing oral functions.

**Conclusion:**

The protocol may enhance oral functionality and reduce hospitalisation duration by systematising multidisciplinary oral management and swallowing rehabilitation, alongside supporting conventional pneumonia prevention strategies.

## Graphical abstract

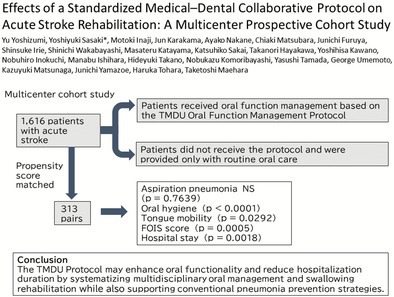

## 1 Introduction

Aspiration pneumonia and other infectious diseases represent common complications associated with acute stroke, potentially resulting in severe consequences, including patient dependency on family or medical facilities and even mortality. Numerous benefits of oral care for patients with acute stroke have been documented. Enhanced oral hygiene practices during the acute phase of artificial ventilation management correlate with a reduction in ventilator‐associated pneumonia (VAP) [1] and a diminished risk of pneumonia onset [2]. Furthermore, dysphagia is a major risk factor for aspiration pneumonia in frail older individuals, particularly in those suffering from cerebrovascular disease [3]. Additionally, a meta‐analysis revealed that stroke patients had poorer overall oral health compared with controls. Further research on screening for oral health issues after stroke should be conducted, and effective management strategies should be developed and implemented [4].

Collaboration between medical and dental professionals has demonstrated effectiveness in improving patient prognosis post‐diagnosis and during rehabilitation [5]. Research indicates that comprehensive oral care, inclusive of dental and oral cleaning, has been shown to be effective for improving the quality of life of inpatients and bedridden individuals in multiple studies [6, 7]. A 2020 Cochrane Library systematic review [8], linked daily oral care for stroke patients with lower onset of pneumonia compared to alternative treatments; however, the review also noted the lack of high‐quality evidence to support customised oral care approaches.

Despite this, insufficient evidence exists regarding the effects of rigorous oral hygiene practices compared to standard cleaning practices in patients with stroke, beyond the prevention of aspiration pneumonia.

Therefore, prior studies have compared the benefits of oral function management for patients with acute stroke in preventing aspiration pneumonia against historical controls. Results indicated the effectiveness of initiating oral function management early (i.e., within 3 days of onset) [9, 10]. Tokyo Medical and Dental University (now the Institute of Science Tokyo) developed the TMDU Oral Function Management Protocol, a multidisciplinary approach involving dental professionals, physicians, nurses and speech therapists (STs). First implemented clinically in 2016 to foster medical–dental collaboration, this protocol aims to standardise oral function management procedures for patients with acute stroke [11]. This protocol is distinctive for its effort to systematise oral management and swallowing rehabilitation, thus allowing for seamless collaboration among a multidisciplinary team comprising physicians, nurses and STs, alongside oral management provided by dental and medical practitioners.

This study thus aims to underscore the significance of early oral function management for patients with acute stroke and to demonstrate the effectiveness of the Protocol in preventing the onset of aspiration pneumonia, as well as its impact on outcomes unrelated to pneumonia. To achieve this, a multicentre collaborative observational study was conducted, comparing cases from multiple hospitals implementing the Protocol against those utilizing conventional oral care techniques to evaluate its effectiveness and to verify its applicability at other medical institutions.

## 2 Methods

### 2.1 Study Design and Setting

**FIGURE 1:**
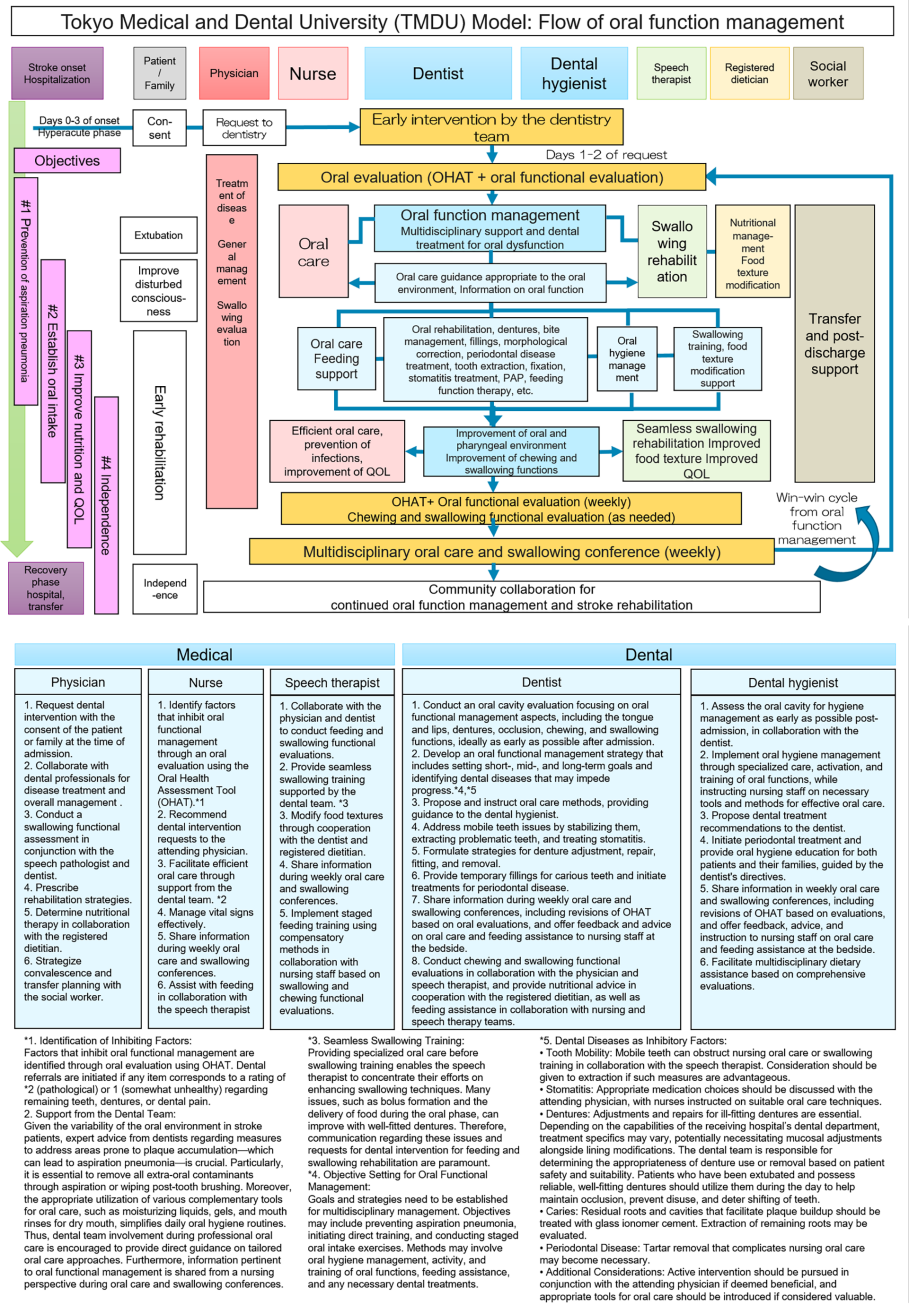
Flow and role of each healthcare professional in the Tokyo Medical and Dental University (TMDU) Oral Function Management Protocol.

This was a prospective multicentre observational cohort study with propensity score matching comparing two real‐world care approaches for acute stroke across 17 affiliated hospitals: of these, 11 facilities implemented a structured multidisciplinary oral function management program based on the TMDU Oral Function Management Protocol [1] (Protocol group) (Figure 1), while six facilities did not follow the protocol and provided only routine (conventional) oral care (control group). Patients with acute stroke admitted to these hospitals between 31 July 2017, and 27 January 2021 were included in the study. No randomisation was performed; allocation to Protocol or control group was determined by the hospital's adoption of the multidisciplinary program, not by investigators or random assignment.

### 2.2 Ethical Considerations

The study was approved by the Ethics Committee of the Institute of Science Tokyo, School of Dentistry (Approval no. D2017‐066). Written or oral (where written was not possible) informed consent was obtained from all patients in the Protocol group; in cases of impaired consciousness, consent was provided by a family member or representative. For the control group, the requirement of individual consent was waived, as procedures fell within standard care, and opt‐out information was made available via hospital posters.

### 2.3 Participants

The following were the inclusion criteria for the study:

- patients with acute stroke;
- patients providing informed consent to participate in the study (Protocol group only); and
- patients commencing oral function management within 7 days of stroke onset.

Patients who fulfilled one or more of the following criteria were excluded from the study:

- patients deemed ineligible by the principal investigator—this included patients in the acute phase whose primary treatment was focused on other conditions, such as malignant tumours as determined by the attending physician;
- patients with presence of pneumonia complications at the time of admission; and
- those in the control group who opted out of having their clinical data used for this observational study.

### 2.4 Intervention Protocol

The intervention was conducted for medical purposes and not for research purposes, and optimal medical care was administered to the patients. In prior studies utilising the previously published TMDU Oral Function Management Protocol [10, 11], oral function management was performed within 3 days of admission. However, since this was a multicentre collaborative study, it was challenging to provide oral function management within that timeframe at all participating institutions in a consistent manner. Consequently, the protocol was modified to permit early oral function management within 7 days of admission.

The elements of the protocol employed in this study are as follows:

- Oral function management for patients with stroke in the hyperacute phase—within 7 days of admission.
- Oral functional evaluation using the Oral Health Assessment Tool (OHAT) [12, 13] and multidisciplinary oral function management by a nurse, ST, and dental professional. A multidisciplinary information‐sharing sheet was utilised to facilitate communication among healthcare professionals (See Appendix S1).
- Weekly multidisciplinary conferences for information sharing and collaboration regarding oral function management and swallowing rehabilitation.
- Frequency of oral function management based on OHAT score:
  - Poor oral health (OHAT score of ≥ 8 points): oral function management by dental professionals ≥ 3 sessions/week, in addition to nurse‐led oral care. ‘Oral care’ refers to the procedures performed by nurses or by the patients themselves; ‘oral function management’ refers to the oral management performed by dental professionals.
  - Fair oral health (OHAT score of 4–7 points): oral function management (2 sessions/week) plus oral care.
  - Good oral health (OHAT score of ≤ 3 points): oral function management (1 session/week) plus oral care.
- Specialised dental treatment and oral function management included:
  - Professional oral prophylaxis and periodontal treatment for poor oral hygiene;
  - Guidance on daily oral care;
  - Conservative dental treatments to avert complications such as inadvertent swallowing of fallen teeth and injuries from sharp teeth;
  - Surgical treatments for mobile teeth and residual roots that could negatively impact oral hygiene and dietary intake;
  - Prosthetic treatments like the repair and adjustment of ill‐fitting dental devices; and
  - Oral prophylaxis and swallowing rehabilitation based on functional evaluations.

The criteria for determining the termination of oral function management were improvement in OHAT score = 0, achievement of independent self‐care, and regular, and stable oral intake (Dysphagia Severity Scale [DSS] = 7 points and Functional Oral Intake Scale [FOIS] = 7 points). If these criteria were not met, oral function management typically continued until discharge.

The primary outcomes were the incidence of pneumonia and aspiration pneumonia, and the secondary outcomes were the length of hospital stay and improvement in oral function. Additional data collected from patient medical records included: (i) basic demographic characteristics, (ii) patient background prior to stroke onset, (iii) admission evaluations, (iv) treatment and rehabilitation details for stroke, (v) pneumonia evaluations, and (vi) discharge evaluations.

### 2.5 Sample Size Calculation

The required sample size for inter‐group comparison post‐matching was established based on pneumonia incidence after 5 days of stroke onset or the primary outcomes determined during the study design phase. According to a previous study [9], the rates were estimated at 14% for the Protocol group and 22% for the control group. Consequently, 306 patients per group were determined to provide adequate statistical power (0.80) and a significance level of 0.05 when employing Fisher's exact test. Due to the unpredictable nature of cases matched through propensity score matching and exploratory analysis requirements for secondary outcomes, an effort was made to maximise the sample size during the study period.

### 2.6 Statistical Analysis

**FIGURE 2:**
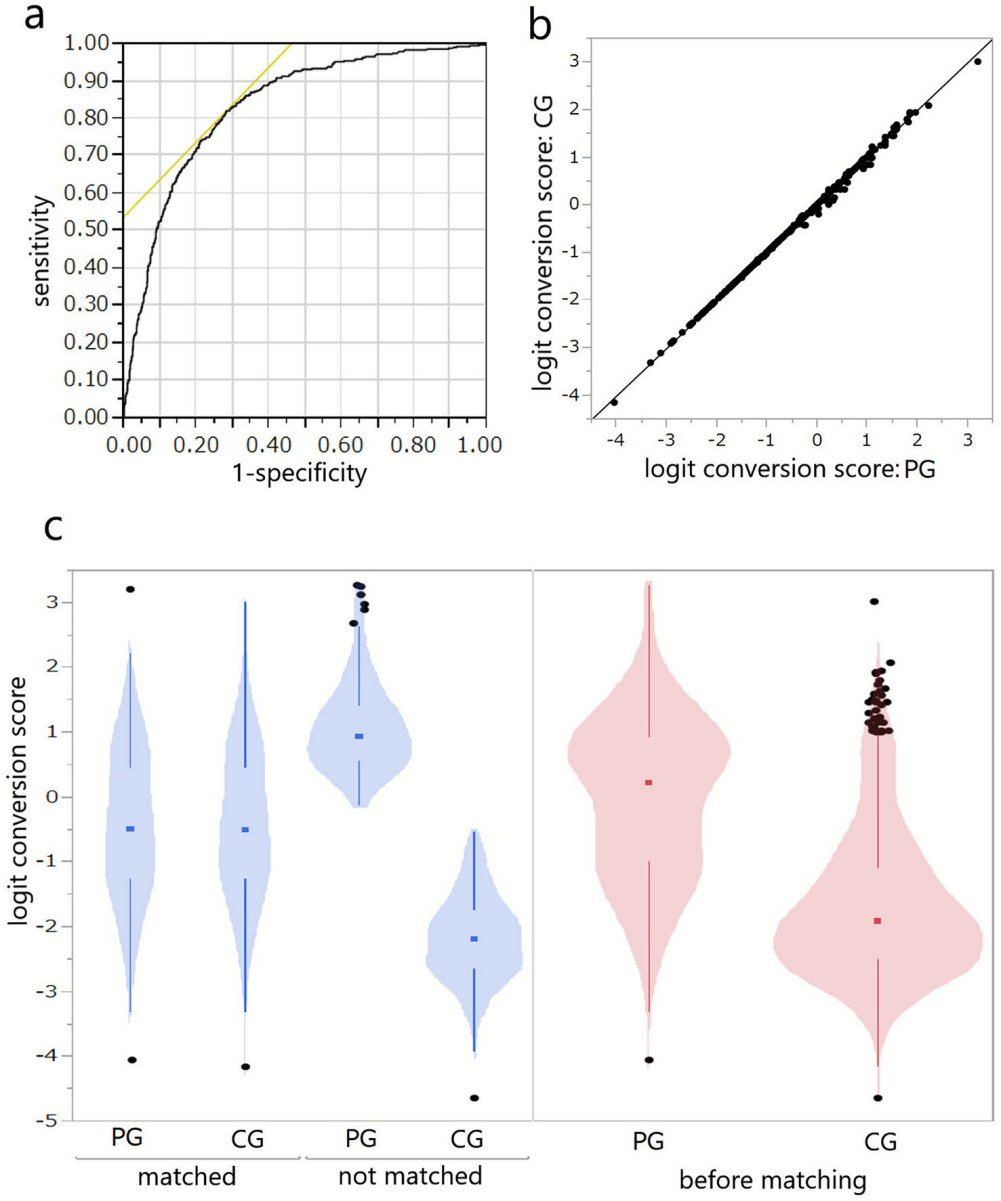
(a): ROC curves for logistic regression analysis for propensity score calculation. The AUC was 0.835. (b): Post‐matching propensity score distribution. (c): Violin plot before/after propensity score matching. PG: Protocol group. CG: Control group.

To reduce confounding, propensity score matching were estimated using logistic regression based on stroke severity and initial complications at admission between the two groups. Using the nearest neighbour method with a calliper coefficient of 0.2 between the Protocol and control groups, the calculated propensity scores were matched, yielding 315 patients from each group. A violin plot illustrating the distribution of the logit‐transformed propensity scores prior to and following matching is shown in Figure 2, which delineates the 630 matched patients. Following the exclusion of two pairs due to missing secondary outcome data, the analysis utilised data from 626 patients (313 pairs). Furthermore, matched patients were analysed for variables related to post‐intervention mortality, pneumonia onset, and quality of life improvement.

Regarding univariate statistics, normally distributed continuous variables were expressed as means and standard deviations and analysed using Welch's *t*‐test for between‐group comparisons. Non‐normally distributed variables, as confirmed by the Shapiro–Wilk test, were expressed as medians with interquartile ranges and analysed with the Mann–Whitney *U* test. Categorical variables, including binary variables, were expressed as frequencies and percentages, with Fisher's exact test applied to examine independence. The Cochran–Armitage test was utilised to assess trends between groups for ordinal variables. The significance level was set at 0.05, with significance probabilities reported. Statistical analysis was conducted using JMP 9.0.3 (SAS Institute Inc., Cary, NC, USA).

## 3 Results

Data from a total of 1616 participants were acquired utilising the aforementioned methods. The mean ages of the Protocol and control groups were 71.6 ± 14.0 years and 72.7 ± 13.6 years, respectively, with no significant differences observed. Significant variations between the two groups were noted for several comorbidities, including congestive heart failure, cerebrovascular disease, chronic lung disease, hemiplegia, solid tumours, smoking status, National Institutes of Health Stroke Scale (NIHSS), thrombolytic therapy (tPA), necessity for surgery, general anaesthesia, intubation, artificial respiration, referrals for speech therapy, referrals for occupational therapy, antiplatelet agent usage, anticoagulation therapy, stroke classification, nutritional intake method, and C‐reactive protein (CRP) levels (Table 1).

**TABLE 1:** Comparison of the Protocol and control groups before matching.

	Protocol group		Control group			
Sex_Male	251	(55.53)	667	(57.30)	0.5188	a
Sex_Female	201	(44.47)	497	(42.70)		
Age	75	(64–82)	74.5	(65–83)	0.3255	b
Charlson Comorbidities index	5	(3–6)	5	(3–6)	0.0363*	b
2011 Revised Comorbidities index (not corrected for age)	0	(0–2)	0	(0–0)	< 0.0001*	b
Comorbidities_Myocardial infarction	18	(3.98)	30	(2.58)	0.1354	a
Comorbidities_Congestive heart failure	19	(4.20)	16	(1.37)	0.0005*	a
Comorbidities_Neurovascular disease	112	(24.78)	222	(19.07)	0.0110*	a
Comorbidities_Dementia	29	(6.42)	96	(8.25)	0.2161	a
Comorbidities_Chronic lung disease	16	(3.54)	7	(0.60)	< 0.0001*	a
Comorbidities_Kidney disease (moderate to severe)	24	(5.31)	55	(4.73)	0.6247	a
Comorbidities_Liver disease (all)	7	(1.55)	34	(2.92)	0.1153	a
Comorbidities_Diabetes (Complications not addressed)	94	(20.80)	225	(19.33)	0.5062	a
Comorbidities_Solid tumours	57	(12.61)	85	(7.30)	0.0007*	a
Comorbidities_Hypertension	271	(59.96)	749	(64.35)	0.1005	a
Comorbidities_Hyperlipidemia	91	(20.13)	209	(17.96)	0.3123	a
Comorbidities_Atrial fibrillation	53	(11.73)	106	(9.11)	0.1126	a
Smoking	65	(14.38)	223	(19.16)	0.0243*	a
Excessive alcohol intake	13	(2.88)	50	(4.30)	0.1858	a
NIHSS total	7	(2–17)	2	(0–7)	< 0.0001*	a
NIHSS~5	177	(39.78)	774	(70.88)	< 0.0001*	c
NIHSS 6~25	207	(46.52)	268	(24.54)		
NIHSS 26~	61	(13.71)	50	(4.58)		
On admission_CFS	7	(5–7)	6	(4–7)	< 0.0001*	b
On admission_GCS_E	4	(3, 4)	4	(4)	< 0.0001*	b
On admission_GCS_V	4	(2–5)	5	(4, 5)	< 0.0001*	b
On admission_GCS_M	6	(5, 6)	6	(6)	< 0.0001*	b
On admission_mRS	4	(4, 5)	3	(1–4)	< 0.0001*	b
Treatment for stroke_tPA	32	(7.08)	45	(3.89)	0.0070*	a
Treatment for stroke_Surgery	171	(37.83)	152	(13.13)	< 0.0001*	a
Treatment for stroke_General anaesthesia	128	(28.32)	118	(10.19)	< 0.0001*	a
Treatment for stroke_Intubation	123	(27.21)	106	(9.15)	< 0.0001*	a
Treatment for stroke_Artificial ventilator	119	(26.33)	88	(7.60)	< 0.0001*	a
Treatment for stroke_Antiplatelet therapy	166	(36.73)	707	(61.11)	< 0.0001*	a
Treatment for stroke_Anticoagulant therapy	106	(23.45)	518	(44.77)	< 0.0001*	a
Rehabilitation for stroke_ST	392	(86.73)	947	(81.78)	0.0171*	a
Rehabilitation for stroke_PT	439	(97.12)	1124	(97.06)	0.9488	a
Rehabilitation for stroke_OT	410	(90.71)	1089	(94.04)	0.0177*	a
Classification of stroke Subarachnoid haemorrhage	77	(17.07)	70	(6.11)	< 0.0001*	a
Classification of stroke Cerebral stroke	223	(49.45)	851	(74.32)		
Classification of stroke Cerebral haemorrhage	151	(33.48)	224	(19.56)		
On admission_Route of nutritional intake Intravenous	247	(54.89)	140	(12.23)	< 0.0001*	c
On admission_Route of nutritional intake Parenteral	30	(6.67)	102	(8.91)		
On admission_Route of nutritional intake Oral and parenteral	9	(2.00)	61	(5.33)		
On admission_Route of nutritional intake Oral with modification	82	(18.22)	287	(25.07)		
On admission_Route of nutritional intake Oral without modification	82	(18.22)	555	(48.47)		
On admission_Alb	4	(3.7–4.3)	4	(3.7–4.3)	0.5888	b
On admission_CRP	0.195	(0.1–0.5)	0.11	(0.1–0.4)	0.0144*	b
At first examination_Remaining teeth (*n*)	21	(9–27)	13	(1–25)	< 0.0001*	b
At first examination_Denture use before admission Not required	151	(42.66)	152	(18.38)	< 0.0001*	b
At first examination_Denture use before admission Necessary and used	135	(38.14)	409	(49.46)		
At first examination_Denture use before admission Necessary but not used	68	(19.21)	266	(32.16)		
Pneumonia evaluation_Pneumonia No	394	(87.36)	1087	−93.79	< 0.0001*	a
Pneumonia evaluation_Pneumonia Yes	57	(12.64)	72	(6.21)		
Pneumonia _Aspiration Pneumonia Yes	41	(71.93)	46	(63.01)	0.2837	a
Pneumonia _Aspiration Pneumonia No	16	(28.07)	27	(36.99)		
Aspiration pneumonia No	411	(90.93)	1118	(96.05)	< 0.0001*	a
Aspiration pneumonia Yes	41	(9.07)	46	(3.95)		

*Note:* a: Fisher's exact test, b: Mann–Whitney *U* test, c: Cochran–Armitage test.

Abbreviations: Alb, albumin; CFS, clinical frailty scale; CRP, C‐reactive protein; GCS_E, glasgow coma scale eyes; GCS_M, glasgow coma scale motor; GCS_V, glasgow coma scale Verbal; mRS, modified rankin scale; NIHSS, national institute of health stroke Scale; OT, occupational therapy; PT, physical therapy; ST, speech therapy; tPA, tissue plasminogen activator.

* *p* < 0.05.

The explanatory variables used to calculate the propensity score by multiple logistic regression analysis included sex, age, presence of comorbidity, smoking, excessive alcohol consumption, use of tPA, need for surgery, general anesthesia, intubation, artificial respiration, rehabilitation, antiplatelet and anticoagulant therapy during stroke treatment, usage of antiplatelet agents, stroke classification, nutritional intake method, serum albumin, CRP levels, and NIHSS score classification at admission. The results of the analysis are displayed in Table 2, revealing significance (*p* < 0.0001), a pseudo coefficient of determination of 0.25, and an area under the curve (AUC) of the receiver operating characteristic (ROC) curve of 0.835.

**TABLE 2:** Multiple logistic analysis results for propensity score calculation.

Factors	Likelihood ratio chi‐square	*p*
Sex	1.96947013	0.1605
Age	2.28452507	0.1307
Comorbidities_myocardial infarction	0.03227745	0.8574
Comorbidities_congestive heart failure	7.99807148	0.0047*
Comorbidities_neurovascular disease	5.38434541	0.0203*
Comorbidities_dementia	2.0430354	0.1529
Comorbidities_chronic lung disease	22.4260716	< 0.0001*
Comorbidities_kidney disease (moderate to severe)	1.78717864	0.1813
Comorbidities_liver disease	7.53320932	0.0061*
Comorbidities_diabetes	1.36415463	0.2428
Comorbidities_solid tumours	1.37117164	0.2416
Comorbidities_hypertension	0.34526258	0.5568
Comorbidities_hyperlipidemia	8.48765699	0.0036*
Comorbidities_atrial fibrillation	0.29093911	0.5896
Smoking	3.43845441	0.0637
Excessive alcohol intake	0.08811617	0.7666
NIHSS classification	18.9722192	< 0.0001*
Treatment for stroke_tPA	1.32898396	0.2490
Treatment for stroke_surgery	3.64708087	0.0562
Treatment for stroke_general anaesthesia	0.46675163	0.4945
Treatment for stroke_intubation	3.08507905	0.0790
Treatment for stroke_artificial ventilator	4.02839064	0.0447*
Treatment for stroke_antiplatelet therapy	6.52003376	0.0107*
Treatment for stroke_anticoagulant therapy	19.1396531	< 0.0001*
Rehabilitation for stroke_ST	8.43531943	0.0037*
Rehabilitation for stroke_PT	1.52817796	0.2164
Rehabilitation for stroke_OT	8.70135613	0.0032*
On admission_classification of stroke	0.44594695	0.8001
On admission_route of nutritional intake	93.816046	< 0.0001*
On admission_serum albumin	3.09919822	0.0783
On admission_CRP	1.60754682	0.2048

*Note:* Significance of regression χ^2^ = 465.71. *p* < 0.0001.

Abbreviations: CRP, C‐reactive protein; NIHSS, national institute of health stroke scale; OT, occupational therapy; PT, physical therapy; ST, speech therapy; tPA, tissue plasminogen activator.

* *p* < 0.05.

No significant differences were observed between the study groups for the 313 pairs in all explanatory variables used to calculate the propensity score (Table 3). However, notable differences were found in baseline variables not included in the propensity score calculations, specifically CRP levels upon admission, the number of remaining teeth and denture usage during the initial examination, as well as OHAT scores concerning oral cleanliness, tongue mobility, and FOIS outcomes at the initial examination (Table 4).

**TABLE 3:** Comparison of the Protocol and control groups after matching (variables used for propensity score calculation).

	Protocol group		Control group		*p*	
Sex_Male	179	(57.19)	176	(56.23)	0.8088	a
Sex_Female	134	(42.81)	137	(43.77)		
Age	75	(66–82)	73	(63–83)	0.6989	b
Comorbidities_Myocardial infarction	13	(4.15)	9	(2.88)	0.3853	a
Comorbidities_Congestive heart failure	8	(2.56)	11	(3.51)	0.4846	a
Comorbidities_Neurovascular disease	80	(25.56)	83	(26.52)	0.7847	a
Comorbidities_Dementia	21	(6.71)	24	(7.67)	0.6425	a
Comorbidities_Chronic lung disease	7	(2.24)	7	(2.24)	1.0000	a
Comorbidities_Kidney disease (moderate to severe)	16	(5.11)	17	(5.43)	0.8581	a
Comorbidities_Liver disease (all)	6	(1.92)	6	(1.92)	1.0000	a
Comorbidities_Diabetes (Complications not addressed)	67	(21.41)	67	(21.41)	1.0000	a
Comorbidities_Solid tumours	35	(11.18)	33	(10.54)	0.7973	a
Comorbidities_Hypertension	192	(61.34)	197	(62.94)	0.6803	a
Comorbidities_Hyperlipidemia	65	(20.77)	71	(22.68)	0.5609	a
Comorbidities_Atrial fibrillation	37	(11.82)	34	(10.86)	0.7053	a
Smoking	47	(15.02)	42	(13.42)	0.5672	a
Excessive alcohol intake	9	(2.88)	6	(1.92)	0.4330	a
NIHSS Total	6	(2–15)	7	(2–18)	0.8374	a
NIHSS~5	136	(43.45)	144	(46.01)	0.6282	c
NIHSS 6~25	144	(46.01)	136	(43.45)		
NIHSS 26~	33	(10.54)	33	(10.54)		
Treatment for stroke_tPA	25	(7.99)	23	(7.35)	0.7639	a
Treatment for stroke_Surgery	91	(29.07)	85	(27.16)	0.5937	a
Treatment for stroke_General anaesthesia	63	(20.13)	62	(19.81)	0.9204	a
Treatment for stroke_Intubation	60	(19.17)	59	(18.85)	0.9189	a
Treatment for stroke_Artificial ventilator	58	(18.53)	56	(17.89)	0.8359	a
Treatment for stroke_Antiplatelet therapy	134	(42.81)	134	(42.81)	1.0000	a
Treatment for stroke_Anticoagulant therapy	87	(27.80)	70	(22.36)	0.1170	a
Rehabilitation for Stroke_ST	267	(85.30)	270	(86.26)	0.7313	a
Rehabilitation for Stroke_PT	306	(97.76)	304	(97.12)	0.6125	a
Rehabilitation for Stroke_OT	287	(91.69)	287	(91.69)	1.0000	a
Classification of stroke Subarachnoid haemorrhage	36	(11.50)	40	(12.78)	0.8664	a
Classification of stroke Cerebral stroke	178	(56.87)	173	(55.27)		
Classification of stroke Cerebral haemorrhage	99	(31.63)	100	(31.95)		
On admission_Route of nutritional intake Intravenous	132	(42.17)	123	(39.30)	0.5574	c
On admission_Route of nutritional intake Parenteral	27	(8.63)	30	(9.58)		
On admission_Route of nutritional intake Oral and parenteral	9	(2.88)	7	(2.24)		
On admission_Route of nutritional intake Oral with modification	69	(22.04)	75	(23.96)		
On admission_Route of nutritional intake Oral without modification	76	(24.28)	78	(24.92)		
On admission_Alb	4.1	(3.7–4.3)	3.9	(3.6–4.2)	0.1294	b
On admission_CRP	0.2	(0.1–0.55)	0.1	(0.1–0.4)	0.0398*	b

*Note:* a: Fisher's exact test, b: Mann–Whitney *U* test, c: Cochran–Armitage test.

Abbreviations: Alb, albumin; CRP, C‐reactive protein; NIHSS, national institute of health stroke scale; OT, occupational therapy; PT, physical therapy; ST, speech therapy; tPA, tissue plasminogen activator.

* *p* < 0.05.

**TABLE 4:** Comparison of the protocol and control groups after matching (variables not used for propensity score calculation).

	Protocol group		Control group		*p*	
Charlson Comorbidities Index	5	(3–6)	5	(3–6)	0.7372	b
2011 Revised Comorbidities Index (Not corrected for age)	0	(0–1)	0	(0–1)	0.8867	b
On admission_CFS	7	(5–7)	7	(5.5–7)	0.9002	b
On admission_GCS_E	4	(3, 4)	4	(3, 4)	0.5077	b
On admission_GCS_V	4	(3–5)	4	(2–5)	0.3964	b
On admission_GCS_M	6	(5, 6)	6	(5, 6)	0.8729	b
On admission_mRS	4	(3–5)	4	(3–5)	0.0214*	b
At first examination_Remaining teeth (n)	21	(8–27)	17	(2–26)	0.0431*	b
At first examination_Denture use before admission Not required	101	(40.89)	34	(17.00)	< 0.0001*	a
At first examination_Denture use before admission Necessary and used	102	(41.30)	93	(46.50)		
At first examination_Denture use before admission Necessary but not used	44	(17.81)	73	(36.50)		
At first examination_Denture use after admission Not required	98	(40.50)	35	(17.50)	< 0.0001*	a
At first examination_denture use after admission necessary and used	83	(34.30)	78	(39.00)		
At first examination_Denture use after admission Necessary but not used	61	(25.21)	87	(43.50)		
On admission_OHAT_Oral cleanliness Healthy	99	(38.98)	134	(58.52)	< 0.0001*	c
On admission_OHAT_Oral cleanliness Changes	114	(44.88)	88	(38.43)		
On admission_OHAT_Oral cleanliness Unhealthy	41	(16.14)	7	(3.06)		
On admission_Tongue exercise Extends beyond the lower lip	172	(67.98)	136	(59.39)	0.7908	c
On admission_Tongue exercise Reaches the lower lip	20	(7.91)	49	(21.40)		
On admission_Tongue exercise Does not reach the lower lip	15	(5.93)	17	(7.42)		
On admission_tongue exercise The lip does not move	46	(18.18)	27	(11.79)		
On admission_FOIS 1–3	97	(38.19)	38	(17.84)	< 0.0001*	c
On admission_FOIS 4–6	98	(38.58)	95	(44.60)		
On admission_FOIS 7	59	(23.23)	80	(37.56)		
On admission_BDR (brushing) Independent	121	(47.64)	87	(37.99)	0.0977	c
On admission_BDR (brushing) requires partial assistance	49	(19.29)	58	(25.33)		
On admission_BDR (brushing) requires total assistance	84	(33.07)	84	(36.68)		
Pneumonia evaluation_Pneumonia No	279	(89.14)	282	(90.10)	0.6943	a
Pneumonia evaluation_Pneumonia Yes	34	(10.86)	31	(9.90)		
Pneumonia _Aspiration Pneumonia Yes	23	(71.88)	25	(86.21)	0.1722	a
Pneumonia _Aspiration Pneumonia no	9	(28.13)	4	(13.79)		
Aspiration Pneumonia no	290	(92.65)	288	(92.01)	0.7639	a
Aspiration Pneumonia yes	23	(7.35)	25	(7.99)		
Number of days from admission to onset of pneumonia	2	(1–5.75)	2	(0–8.5)	0.9477	b
Number of days from admission to onset of pneumonia Aspiration pneumonia yes	3	(1–8)	2	(0–5.5)	0.2184	b
Days from admission to medical/dental evaluation at discharge	18	(13–26)	27	(16–42.5)	< 0.0001*	b
Hospital stay (days)	21	(15–31.5)	27	(16–42.5)	0.0018*	b
Death	8	(2.56)	18	(5.75)	0.0452*	a
At discharge_CFS	6	(4–7)	6	(4–7)	0.8843	b
At discharge_GCS_E	4	(4)	4	(4)	0.4742	b
At discharge_GCS_V	5	(4, 5)	5	(4, 5)	0.0420*	b
At discharge_GCS_M	6	(6)	6	(6)	0.3792	b
At discharge_mRS	4	(2–4)	3	(2–4)	0.1822	b
At discharge_Route of nutritional intake Intravenous	5	(1.64)	5	(1.61)	0.1426	c
At discharge_Route of nutritional intake Parenteral	36	(11.80)	22	(7.07)		
At discharge_route of nutritional intake Oral and parenteral	30	(9.84)	27	(8.68)		
At discharge_route of nutritional intake Oral with modification	83	(27.21)	98	(31.51)		
At discharge_route of nutritional intake Oral without modification	151	(49.51)	159	(51.13)		
Alb (At discharge)	3.4	(3.1–3.8)	3.5	(3.0–3.9)	0.1745	b
CRP (At discharge)	0.265	(0.1–0.8325)	0.215	(0.1–1.325)	0.8210	b
At discharge_OHAT_Oral cleanliness Healthy	177	(59.40)	179	(67.55)	0.1692	c
At discharge_OHAT_Oral cleanliness Changes	111	(37.25)	73	(27.55)		
At discharge_OHAT_oral cleanliness Unhealthy	10	(3.36)	13	(4.91)		
At discharge_Tongue exercise extends beyond the lower lip	226	(75.84)	168	(63.88)	0.0148*	c
At discharge_Tongue exercise reaches the lower lip	29	(9.73)	47	(17.87)		
At discharge_Tongue exercise does not reach the lower lip	23	(7.72)	20	(7.60)		
At discharge_Tongue exercise the lip does not move	20	(6.71)	28	(10.65)		
At discharge_FOIS 1–3	58	(19.40)	45	(15.90)	0.0035*	c
At discharge_FOIS 4–6	127	(42.47)	89	(31.45)		
At discharge_FOIS 7	114	(38.13)	149	(52.65)		
At discharge_BDR(brushing) Independent	188	(63.51)	132	(49.81)	0.0040*	c
At discharge_BDR(brushing) Requires partial assistance	48	(16.22)	62	(23.40)		
At discharge_BDR(brushing) Requires total assistance	60	(20.27)	71	(26.79)		
Difference (before/after)_OHAT_oral cleanliness Improvement	88	(36.21)	41	(18.55)	< 0.0001*	c
Difference (before/after)_OHAT_oral cleanliness No change	138	(56.79)	154	(69.68)		
Difference (before/after)_OHAT_oral cleanliness Deterioration	17	(7.00)	26	(11.76)		
Difference (before/after)_Tongue exercise Improvement	54	(22.31)	48	(21.72)	0.0292*	c
Difference (before/after)_Tongue exercise No change	179	(73.97)	142	(64.25)		
Difference (before/after)_Tongue exercise Deterioration	9	(3.72)	31	(14.03)		
Difference (before/after)_FOIS Improvement	119	(48.97)	68	(33.50)	0.0005*	c
Difference (before/after)_FOIS No change	110	(45.27)	113	(55.67)		
Difference (before/after)_FOIS Deterioration	14	(5.76)	22	(10.84)		
Difference (before/after)_BDR(brushing) Improvement	69	(28.63)	74	(33.18)	0.5273	c
Difference (before/after)_BDR(brushing) No change	167	(69.29)	127	(56.95)		
Difference (before/after)_BDR(brushing) Deterioration	5	(2.07)	22	(9.87)		

*Note:* a: Fisher's exact test, b: Mann–Whitney *U* test, c: Cochran–Armitage test.

Abbreviations: BDR, BDR index, assessment of independence for brushing, denture wearing, mouth rinsing; CFS, clinical frailty scale; FOIS, functional oral intake scale; GCS_E, glasgow coma scale eyes; GCS_M, glasgow coma scale motor; GCS_V, glasgow coma scale verbal; mRS, modified rankin scale; OHAT, oral health assessment tool.

* *p* < 0.05.

The incidence of pneumonia onset, regarded as the primary outcome, was 10.86% in the Protocol group compared to 9.90% in the control group, without a significant difference (*p* = 0.6943). No significant difference was observed in the incidence of aspiration pneumonia (*p* = 0.7639). Conversely, a significant difference was detected in the rate of in‐hospital mortality between the Protocol group (2.6%) and control group (5.8%) (*p* = 0.045).

Among the secondary outcomes, significant differences were observed in the median number of days from admission to the discharge evaluation (Protocol group, 18 days; control group, 27 days; *p* ≤ 0.0001) and in the median hospital stay (Protocol group, 21 days; control group, 27 days; *p* = 0.002).

In terms of oral function, significant differences were observed in changes in oral cleanliness between the first examination and discharge evaluation, indicating improvements in oral hygiene in a higher percentage of patients in the Protocol group. Likewise, a significantly higher percentage in the Protocol group exhibited improvements in tongue mobility and FOIS or maintained their status without deterioration between the initial examination and discharge evaluation. However, no significant differences were observed between the two groups regarding changes in independence in tooth brushing behaviors following the intervention.

## 4 Discussion

This was the first multicentre prospective cohort study to investigate the impact of early oral function management for patients with acute stroke, employing a highly reliable research design that mitigates confounding factors related to patients' baseline severity through propensity score matching. Despite significant differences in baseline variables impacting prognosis between the two groups prior to matching (Table 1), the derived propensity score effectively differentiated between the groups (Table 2, *p* < 0.0001), with no substantial variations post‐matching for most potential confounding variables (Table 3). Therefore, the application of propensity score matching in this research is deemed appropriate.

The development of pneumonia, identified as the main outcome of this study, merits discussion. The incidence of aspiration pneumonia in patients experiencing dysphagia due to acute stroke is estimated to range between 13%–33% [2], while pneumonia linked to oral care is reported at 1%–7% [1, 2]. This aligns with the pneumonia onset rate observed in the control group, thereby supporting the notion that conventional dental and oral hygiene practices remain effective within that group. This effectiveness is likely supported by a 1999 study articulating the benefits of meticulous oral hygiene in preventing aspiration pneumonia [14], and the increased adherence to oral hygiene protocols established by nursing staff since then.

The length of time from admission to discharge evaluations and hospital stays, both presumed to correlate with the primary outcome, were markedly shorter in the Protocol group. These outcomes are attributed to the advantages of the Oral Function Management Protocol implemented in this study, which standardises procedures allowing for the seamless early oral function management and swallowing rehabilitation conducted by a multidisciplinary team comprising physicians, nurses, and STs, in addition to oral management by dental professionals. The utilisation of this Protocol throughout the hospital stay suggests validated effects across multiple care centres. A preceding study [15] reported that early oral care conducted by a multidisciplinary team in conjunction with early mobilisation significantly diminished the incidence of stroke‐related pneumonia within 7 days, as well as the percentages of patients requiring medical treatment subsequent to pneumonia recurrence after stroke. Numerous additional studies have similarly indicated that perioperative oral management has been effective in reducing hospital stays across various diseases, including during the peri‐operative period for malignancies [16, 17].

Significant differences in changes in OHAT oral hygiene post‐intervention were observed; a larger proportion of Protocol group participants experienced improvement. This enhancement can likely be attributed to the following reasons: first, while nurses predominantly managed oral care in the control group, dental professionals provided oral function management in the Protocol group, thereby increasing the frequency of care; and second, beyond routine oral care, the dental team also delivered comprehensive oral management, including emergency dental treatments. Additionally, a markedly higher percentage of patients in the Protocol group showed improvements or at least stable outcomes in tongue mobility and FOIS scores. This improvement is likely associated with the oral function management that enhanced oral functionality and mitigated swallowing disorders through the restoration of bite support. These findings align with a post‐study report in which multivariate analysis using improvements in FOIS scores as the dependent variable demonstrated associations between improvement in nutritional intake during acute care and the number of functional teeth, DSS, and OHAT values. This highlights the critical connection between the recovery of swallowing function and overall oral health for patients with stroke in resuming oral intake [18]. It has also been noted that patients facing initial challenges with tongue function, denture fit, and oral hygiene, as assessed by the OHAT at admission, require more intensive oral function management [19]. Furthermore, a report underscored the importance of augmenting oral health evaluations for patients with stroke, adapting individual healthcare education and management strategies for high‐risk groups, and advocating for oral health [20].

The observed improvement in oral indicators, particularly those related to oral function, is likely attributable to the early aggressive oral function management supplied by dental and rehabilitation professionals tailored to patient symptoms. Furthermore, this underscores the effective design of the Protocol as an integrated care package. However, there were no changes in the degree of independence in tooth brushing behaviours between the two groups following oral function management. This outcome may be explained by the provision of oral care by medical professionals for both groups; thus, an amendment to the Protocol may be warranted, focusing on promoting patient independence through rehabilitation efforts led by physiotherapists or occupational therapists. The present Protocol is anticipated to necessitate more advanced skills from dental professionals and rehabilitation specialists compared to conventional dental and oral hygiene practices. However, with suitable training, it has been established that care can be effectively delivered without the exclusive involvement of a dental professional. Subsequent research could prove vital in elucidating the mechanisms underpinning the reduction in hospital stays attributed to specialised care, as observed in other medical conditions.

One limitation of this study is the absence of randomization in participant allocation, which may introduce selection bias and confounding variables. Without random assignment, differences between groups could have influenced the outcomes independently of the intervention itself. Patients were allocated based on the type of healthcare facilities to which they were admitted: those providing oral function management by specialists versus those lacking such services. Consequently, the patient populations in the Protocol group were predominantly drawn from university hospitals, whereas the control group predominantly comprised patients from municipal hospitals. Nevertheless, it is important to recognise that affiliation with a dentistry department does not guarantee accessibility to oral function management specifically for patients with stroke, as numerous hospitals with dental professionals still confront challenges related to multidisciplinary cooperation between dental and other medical departments [21]. An additional limitation is that the timing of the oral function management intervention varied across institutions, which may have affected its impact. This heterogeneity in onset could lead to inconsistencies, potentially influencing the observed effects.

During the study design phase, we recognized potential admission data disparities between Protocol and control groups, which could introduce confounding variables. Anticipating treatment differences based on stroke severity and care‐seeking behaviour (patients sought care either through direct visits or ambulance transport), we used propensity score matching on clinical variables. Patients who were outliers in each group were excluded from the analysis based on the results of matching. Furthermore, while participants in the Protocol group received oral function management, we do not assume they were fully aware of its intended effects. Nonetheless, the awareness of receiving care may have influenced their behaviour or engagement, which could have contributed to the outcomes. This potential influence should be considered when interpreting the results.

As this was a multicentre study, personal calibration using patients was not feasible, limiting data accuracy. Furthermore, the resources that could be allocated to patients varied across facilities, resulting in missing data for certain items. Nevertheless, the validation of the effectiveness of the Protocol package suggests the necessity of re‐evaluating individual elements to further enhance the effectiveness of the overall care package.

## 5 Conclusions

A key feature of the TMDU Oral Function Management Protocol is its systematised approach, which enables not only dental professionals but also physicians, nurses, and STs to initiate oral function management and dysphagia rehabilitation from an early stage. In addition to the preventive effects of conventional oral care against aspiration pneumonia, the protocol's structured, multidisciplinary early intervention may contribute to shorter hospital stays and improved oral function. Furthermore, a streamlined system for sharing critical information among professionals has been established using a dedicated information‐sharing sheet, enhancing multidisciplinary collaboration.

## Author Contributions

Motoki Inaji, Junichi Furuya, Masateru Katayama, Haruka Tohara and Taketoshi Maehara conceived and designed the study. Yu Yoshizumi, Yoshiyuki Sasaki, Jun Karakama, Ayako Nakane, Chiaki Matsubara, Junichi Furuya, Shinsuke Irie, Shinichi Wakabayashi, Masateru Katayama, Katsuhiko Sskai, Takanori Hayakawa, Yoshihisa Kawano, Nobuhiro Inokuchi, Manabu Ishihara, Hideyuki Takano, Nobukazu Komoribayashi, Yasushi Tamada, George Umemoto, Kazuyuki Matsunaga, and Junichi Yamazoe participated in data acquisition and statistical analysis. Yu Yoshizumi, Yoshiyuki Sasaki, and Motoki Inaji prepared the original draft. Yu Yoshizumi, Yoshiyuki Sasaki, Haruka Tohara, and Taketoshi Maehara reviewed and edited the text. All authors have read and agreed to the published version of the manuscript.

## Funding

This work was supported by Japan Agency for Medical Research and Development, 16768752.H28‐30.

## Conflicts of Interest

The authors declare no conflicts of interest.

## Supporting information


**Appendix S1.** Supporting Information.

## Data Availability

The data that support the findings of this study are available on request from the corresponding author. The data are not publicly available due to privacy or ethical restrictions.
