# Supplementary material for: Effects of a Standardised Medical–Dental Collaborative Protocol on Acute Stroke Rehabilitation: A Multicentre Prospective Cohort Study
Source: J Oral Rehabil. 2026 Jan 2;53(4):866–79. doi: 10.1111/joor.70143 (PMC12980049; doi:10.1111/joor.70143)
Supplement: Supplementary file 1 — Appendix S1. Supporting Information. [file JOOR-53-866-s001.pdf]

Supplementary Material

Appendix A. Multidisciplinary Information Sharing Sheet (OHAT Sheet)

Oral Function Management (Oral Care)
Multidisciplinary Information Sharing Sheet (OHAT Sheet)

	Mon.	Tue.	Wed.	Thu.	Fri.
Oral function management	☐	☐	☐	☐	☐
Assessment	☐	☐	☐	☐	☐

ST rehabilitation ☐ Yes ☐ No

Name	

Date	/	/	/	/
JCS				
Tongue exercise				
DSS				
FOIS				
OHAT				
Lips				
Tongue				
Gums and tissue				
Saliva				
Natural teeth				
Dentures				
Oral cleanliness				
Dental pain				

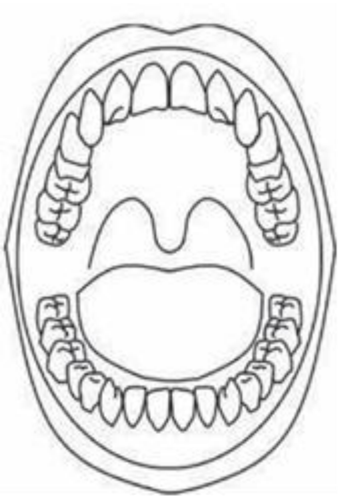

Oral care items	☐Toothbrush	☐Mucosal brush	☐Interdental brush	☐Tongue brush
	☐Sponge brush	☐Oral moisturizers	☐Oral care tissues	☐Others
Oral moistening before care	☐Need		Wiping after care	☐Need
Dentures	☐Recommended for daytime use	☐Recommended for use during mealtimes only	☐Recommended for not use	☐Recommended for use at night
Goal	☐Prevention of aspiration pneumonia	☐Start of direct training	☐Continuation of gradual feeding training	☐Regular oral intake
Oral function management policy	☐Oral hygiene management	☐Oral function activation and training	☐Mastication and swallowing training, dietary support	☐Dentures and dental treatment

Please return this sheet when you are discharged from the hospital.

Ver20170731 Tokyo Medical and Dental University Hospital Department of Swallowing Rehabilitation
